# Library resource sharing and the Medical Library Center of New York*

**DOI:** 10.5195/jmla.2020.854

**Published:** 2020-01-01

**Authors:** Patricia E. Gallagher

**Affiliations:** Librarian, National Information Center on Health Services Research and Health Care Technology, National Library of Medicine, Bethesda, MD, patriciaegallagher@gmail.com

## Abstract

The creation of the Medical Library Center of New York (MLCNY) was a significant contribution to the history of health sciences librarianship as a model for cooperative, democratic, and practical solutions to the issues of storage and resource sharing. The MLCNY’s founding director, Erich Meyerhoff, was a key figure in the successful start-up and ongoing operations of the center, which operated from 1960–2003 and served the greater New York area and beyond. This essay traces the evolution of the center including the creation of the Union Catalog of Medical Periodicals and the demise of the center occasioned by changes in scholarly publishing, technology, and constituent needs.

New York City and its environs have a large number of major health sciences libraries, all located within a 40-mile radius: 7 medical schools, several major research institutes, and an extensive medical society library. In a 1967 article, McCormack estimated that in New York City, there were 7 medical schools; 156 hospitals (in the 5 boroughs, with another 68 in Nassau, Suffolk, and Westchester counties); 18,600 physicians (with another 4,000 in the adjacent counties); and 10% of all internships. McCormack also estimated that 12.8% of all residencies in the United States were located in New York City [1].

By the 1950s, all were facing the same problem: too much material, and not enough space to house it. Expansion of the library space of each individual institution was not an option; another solution needed to be developed. There was an appreciation that older and rarely used materials needed to be kept, but they did not need to be immediately at hand; remote storage of some kind was a consideration, but at what cost, and did all of these libraries really need duplicate remote storage of the same older and/or rarely used materials?

A meeting in March of 1958 brought together over 100 librarians, physicians, and administrators to develop a depository library, one that would allow all cooperating libraries equal access to the materials that it housed [2]. Based on an arrangement that had been developed by the Midwest Inter-Library Corporation—a cooperative agreement developed by 10 university libraries in Illinois, Indiana, Iowa, Kansas, Michigan, and Minnesota—the concept of the Medical Library Center of New York (MLCNY) was developed: a facility that would be the “library for libraries” [3, 4].

One of the advocates for the plan was Erich Meyerhoff, then the librarian of the Downstate Medical Center. Sometime after that meeting, Erich was selected as the founding director of the MLCNY when the institution was incorporated in 1959 [5]. Nine founding partners—the New York Academy of Medicine, Cornell University Medical College, College of Physicians and Surgeons of Columbia University, Memorial Sloan-Kettering Institute for Cancer Research, Albert Einstein College of Medicine, New York University School of Medicine, New York Medical College, the Rockefeller Institute, and the Health Department of the City of New York—and a grant from the John A. Hartford Foundation provided the initial funding [6].

A building was purchased; an 8-story former garage was renovated—even with a building that could support the weight of cars, it was still necessary to increase the floor loads to support the weight of the paper that the MLCNY would be storing. The MLCNY collection was housed on 2 of the floors, and the rest of the space was rented out, providing further income to the project. Each of the 9 founding partners paid a yearly membership fee of $12,000, and the deans or chief executive officers (CEOs) of the participants formed the Board of Trustees. A charter was developed that outlined the goals and mission of the MLCNY, the extent of the collection, and the ways of disseminating the information to the participating institutions. This charter outlined both the book storage and service activities of MLCNY, including the development of a union list and systems of cataloging; acquisition and sharing of books and periodicals; and facilities for research in medical library practice and administration [2].

The MLCNY, according to Erich, needed to be more than just a storehouse of material; it had to provide quick access to the materials it contained. The Midwest Inter-Library Corporation had relied on the US Postal System for delivery of articles. Using the Hampshire Interlibrary Center as an example, Erich knew that retrieval and distribution needed to be faster than the two to three days that normal postal delivery would provide [7]. The geography of the participants also helped: when the MLCNY opened, all of the cooperating libraries were within twenty miles of each other. A daily truck delivery system was initiated. It was possible, since the driver’s route was precisely scheduled and known by all participants, to actually order and receive the paper article in the same day, merely by asking the librarian before you on the truck route to make certain it was included in the day’s pickup.

The venture was not without problems. While some libraries happily divested themselves of older materials, others were less inclined to contribute materials to MLCNY, leaving journal runs with huge gaps and an uneven collection. These libraries often availed themselves of storage space at MLCNY but would not contribute their materials, resulting in more collection duplication, as well as making some items not really accessible to anyone but the storage owner.

But by 1968, the MLCNY had added two more medical schools as sponsoring institutions and eleven hospitals as participating members. The MLCNY collection included journals, monographs, government documents, and medical dissertations. MLCNY subscribed to journals in *Index Medicus* that were not held by area libraries. Monographs consisted of older materials that area libraries generally needed to discard and that might be required on a less frequent basis [8].

The second goal of MLCNY was more complicated: to give members an easy way to determine the vast holdings of the MLCNY and of the participating libraries. When the MLCNY opened, it was determined that, once MLCNY’s holdings were cataloged, the cards representing the holdings would need to be copied and compiled into a printed book format.

With another grant from the Hartford Foundation, MLCNY proceeded to compile the Union Catalog of Medical Periodicals (UCMP), which included not only the holdings of the MLCNY, but of “all pertinent collections in the New York Metropolitan area” [2]. Any contributor to the UCMP received a copy of the published holdings, greatly increasing their ability to find needed materials. The ultimate goal of the project was to enable libraries to find any citations from the MEDLARS (later MEDLINE) system.

In 1966, the first edition of the UCMP was published. Erich and Jacqueline Felter arranged to get computer time, and punch cards of all the libraries’ collections were incorporated into electronic format [9]. This enabled staff to be able to print the holdings of the entire UCMP or just print the holdings of specific libraries. Holdings were entered down to the journal issue level, a novel concept in a period where most union catalogs (like OCLC) only listed ownership of the journal title. Its holdings included the journal collections of sixty-seven participants, fifteen of which were hospital libraries [10].

The programs that ran UCMP also helped other local and regional libraries to create their own union lists. Libraries received their copies of the UCMP on microform sheets, allowing them to quickly locate the best—and often the cheapest—library to supply the article that their client required. By 1979, UCMP was the official regional union list for the New York and New Jersey region of the Regional Medical Libraries (RMLs) in SERHOLD, the National Library of Medicine’s database of serial holdings, making it an essential communication tool for libraries to inform their colleagues of their holdings [11]. Since SERHOLD did not go down to the issue level at this time, the UCMP reports to its participants were still a crucial tool for discovery: if a library needed to quickly find a specific article of a rarely held journal, the microform allowed the library to direct its request more accurately.

As the 1990s began, so too did the Internet and electronic mail. The RML office had signed an interagency agreement with NASA to gain access to NASA’s LIFENET system [12]. This access would enable libraries to have electronic mail for the first time. MLCNY worked with the RML to distribute passwords to its members, allowing librarians from each of the member libraries to quickly communicate with one another [13]. LIFENET also provided Internet access to these libraries for the first time. Though rudimentary when compared with the power of the Internet today, at the time, it greatly enhanced communication in the region—and the country. Thus, when UCMP Online™ was launched in 1996, libraries in the MLCNY system already had the Internet access required to use it. Now accessible from reference desks rather than requiring a microform reader to access it, UCMP grew beyond merely a location tool: it became a reference tool that allowed librarians to answer questions about journal titles and their history [10].

The closure of the MLCNY was brought about by a number of factors involving space, money, and technology. First, the space in which it was housed, on 102nd Street, adjacent to Mount Sinai Medical Center, became prime real estate. The hospital was expanding, and the building would be an attractive addition to their holdings. While hospital libraries still appreciated the value provided by their dollars, some of the supporting libraries did not. As previously mentioned, some had not divested themselves of their collections—those libraries could simply find another storage facility, potentially at a lower price for any items housed at MLCNY. They also no longer required the interlibrary loan functions that MLCNY provided when the project was initiated in the 1950s. Finally, the Internet was making the truck delivery quaint. Telefacsimile (“fax”) had become a tool in the 1980s, but by 2003, the Internet was making article delivery faster and more convenient. Plus, it was becoming easier to obtain journal articles directly from the publishers. It was costly, but in cases where emergency use of a single journal article was required, it was quicker and more economical to pay a fee once rather than subscribe to an interlibrary loan delivery system.

UCMP had also become unnecessary. By 1998, SERHOLD allowed libraries to independently update their own holdings in the system [14]. With DOCLINE locating to the issue level, it was no longer necessary to use UCMP in advance, either via microform or online, to get a speedy response. A library merely needed to place its request in the system, and DOCLINE would locate a holding institution, based on the institution’s routing table. Without viable products to vend and without the support of the sponsoring organizations, MLCNY’s demise was a foregone conclusion.

Never let it be said that the need for resource sharing has disappeared. It is, in fact, as necessary in the Internet age as it was in the paper era. Certainly, it is easier to get instant access to single articles than it was in 1959, but prices are often beyond the reach of most libraries. Vendors control access to journals, with prices escalating on a yearly level. According to *Library Journal*, the cost for health sciences titles increased by 9% between 2018 and 2019 [15], and the Association of Research Libraries estimates that, between 1986 and 2014, the cost of journals and other recurring materials increased by 521%, while the consumer price index increased by 118% [16]. Libraries increasingly can only purchase electronic access, which perforce means the content resides on a publisher website and publishers’ contracts place limits on whom the library can let use the articles. Journals are no longer always purchased individually, but in bundles—often resulting in subscriptions to unwanted journals, wasting library time and funds.

New cooperative groups have been formed to assist libraries. One example is the Group Licensing Initiative (GLI) of the Health Sciences Library Association of New Jersey [17]. Participation in the GLI encompasses libraries in twenty states, the District of Columbia, and the US Virgin Islands, and offers participants access to individual electronic journal titles and collections at negotiated group savings. This and other group arrangements help the librarian, whether in a large university setting or in a one-person library, traverse the rocky road of getting information to their clientele.

It is telling that, when asked to participate in the Medical Library Association oral history program, Erich consented with the proviso that the history be not about himself, but about the Medical Library Center of New York as MLCNY “fulfill[ed] some of his own social beliefs” [9]. In the end, it was a dream that died and a loss he always mourned [18]. But it is a dream that is being reborn in new and exciting forms. For librarians, it remains a symbol of what libraries should be: a means of putting the client first and of providing information in a timely and democratic way.

**Patricia E. Gallagher, AHIP, FMLA,** patriciaegallagher@gmail.com, Librarian, National Information Center on Health Services Research and Health Care Technology, National Library of Medicine, Bethesda, MD

## References

[1] McCormack JE The New York City story. Bull NY Acad Med.

[2] Gregersen MI (1958). A plan for a cooperative medical library center in New York. Bull NY Acad Med.

[3] Meyerhoff E (1963). The Medical Library Center of New York: an experiment in cooperative acquisition and storage of medical library materials. Bull Med Libr Assoc.

[4] Center for Research Libraries History of CRL [Internet].

[5] (1962). The Medical Library Center of New York. Bull NY Academy Med.

[6] Annan GL, Felter JW, Meyerhoff E, Ash L (1964). Regional plans for medical library service: New York State and New York metropolitan area. Bull Med Libr Assoc.

[7] Luddington FB (1952). Hampshire Inter-Library Center. ALA Bull.

[8] Felter JW (1968). The Medical Library Center of New York: a progress report. Bull Med Libr Assoc.

[9] Meyerhoff E Anderson RK, interviewer. Medical Library Association oral history interview about the Medical Library Center of New York. Oral history conducted 7 Aug 2003.

[10] Dempsey R, Weinstein L (1999). UCMP and the Internet help hospital libraries share resources. Bull Med Libr Assoc.

[11] Battistella MS (1991). The OCLC®-SERHOLD® connection: an evolution in health sciences union listing. Bull Med Libr Assoc.

[12] Speaker SL (2018). An historical overview of the National Network of Libraries of Medicine, 1985–2015. J Med Libr Assoc.

[13] Weinstein L (1994). LIFENET/INTERNET and the health science librarian. Spec Libr.

[14] National Library of Medicine (1998). MLA 1998. NLM Tech Bull [Internet].

[15] Bosche S, Albee B, Romaine S (2019). Deal or no deal | periodicals price survey 2019. Libr J [Internet].

[16] Resnick B, Belluz J (2019). The war to free science: how librarians, pirates, and funders are liberating the world’s academic research from paywalls. Vox [Internet].

[17] Health Sciences Library Association of New Jersey Group licensing [Internet].

[18] Meyerhoff E (2004). Death in the family: the Medical Library Center of New York, 1960–2003. Bull Med Libr Assoc.

